# Metabolic syndrome as an independent risk factor for glaucoma: a nationally representative study

**DOI:** 10.1186/s13098-023-01151-5

**Published:** 2023-08-24

**Authors:** Jun-Hyuk Lee, Yu-Jin Kwon, Sung Jin Kim, Boyoung Joung

**Affiliations:** 1 Department of Family Medicine, Nowon Eulji Medical Center, Eulji University School of Medicine, Seoul, 01830 Republic of Korea; 2 Department of Medicine, Graduate School of Hanyang University, Seoul, 04763 Republic of Korea; 3 Department of Family Medicine, Yongin Severance Hospital, Yonsei University College of Medicine, Yongin, 16995 Republic of Korea; 4 Department of Ophthalmology, Nowon Eulji Medical Center, Eulji University School of Medicine, 68 Hangeulbiseok-ro, Nowon-gu, Seoul, 01830 Republic of Korea; 5 Division of Cardiology, Department of Internal Medicine, Yonsei University College of Medicine, 50, Yonsei-ro, Seodaemun-gu, Seoul, 03722 Republic of Korea

**Keywords:** Metabolic syndrome, Insulin resistance, Glaucoma, Korea

## Abstract

**Background:**

Central insulin resistance contributes to glaucoma development. Given the close association between metabolic syndrome MetS and insulin resistance, this study aimed to determine whether MetS is associated with glaucoma risk.

**Methods:**

We analyzed data from 11,499 adults aged ≥ 19 years in the 2019–2021 Korean National Health and Nutrition Examination Survey and applied sampling weights to represent the general Korean population. Participants were classified into groups with or without MetS. Ocular hypertension (HTN) was defined as intraocular pressure > 21 mmHg. Primary open-angle glaucoma (POAG) was diagnosed based on the results of a visual field test and optical coherence tomography using the criteria published by the International Society for Geographic and Epidemiological Ophthalmology. We further divided POAG into normal tension (NTG) and POAG with ocular HTN. A spline curve was drawn to determine the dose–response relationship between the number of MetS components and risk of POAG. Odds ratios (ORs) with 95% confidence interval (CI) for POAG according to MetS status were estimated using weighted logistic regression analyses.

**Results:**

The prevalence of POAG was 5.7% and 3.5%, respectively, in groups with and without MetS. We identified a dose–response relationship between the number of MetS components and risk of POAG. Unadjusted ORs (95% CI) for POAG in the group with MetS was 1.85 (1.52–2.25), compared with those without MetS. The trends persisted in adjusted models. The fully-adjusted OR (95% CI) for POAG was 1.47 (1.04–2.09) in the group with MetS. Subgroup analysis revealed that a significant relationship remained only in the NTG group (fully adjusted OR, 1.50; 95% CI 1.05–2.15).

**Conclusions:**

A comprehensive ophthalmological assessment should be considered for persons with MetS who are at increased risk of POAG, particularly NTG.

**Supplementary Information:**

The online version contains supplementary material available at 10.1186/s13098-023-01151-5.

## Background

Glaucoma is characterized by the progressive loss of retinal ganglion cells (RGCs) and optic nerve degeneration that leads to irreversible vision loss [1]. An estimated 3.6 million people aged ≥ 50 y had glaucoma during 2020, which rendered it the second-most prevalent cause of vision loss after cataracts [2]. Based on the global burden of diseases 2019 [3], the prevalence of glaucoma has steadily increased from 3,881,624 in 1990 to 7,473,400 in 2019. The overall prevalence of primary open-angle glaucoma (POAG) in Asia is 1.1%–3.9%, of which normal tension glaucoma (NTG) comprises 46.9%–92.3% [4]. Impaired vision due to glaucoma causes economic losses worldwide; 30.2% of ~ 160.7 million employees with impaired vision, lost their jobs [5]. In 2018, the estimated global economic loss attributed to vision impaired by glaucoma was $410.7 billion in purchasing power parity [5]. Risk factors for glaucoma comprise intraocular pressure (IOP), thinner central corneal thickness, higher cup-to-disk ratios of the optic disc, age, sex, race, family history of glaucoma, smoking, obstructive sleep apnea, diabetes mellitus, hypertension (HTN), and hypotension [6, 7]. Accumulated evidence suggests that glaucoma shares some pathophysiological mechanisms with neurodegenerative diseases that involve central nervous system (CNS) damage and neuroinflammatory states [8, 9]. In particular, central insulin signaling dysfunction can lead to transsynaptic neurodegeneration [10]. In fact, a proposed brain diabetes theory considers glaucoma as diabetes type 4 [11, 12].

Metabolic syndrome (MetS) is a group of metabolic disorders characterized by excessive visceral fat, high blood glucose, high blood pressure, and atherogenic dyslipidemia [13]. The worldwide prevalence of MetS is steadily increasing, and it leads to various health complications including elevated risk for ocular HTN and glaucoma [14]. Insulin resistance might be a link between MetS and glaucoma, considering the close association between MetS and insulin resistance. Relationships among MetS, glaucomatous optic neuropathy, and ocular HTN have been explored in patients with glaucoma [15–18]. However, these studies were limited by sole reliance on the International Classification of Diseases (ICD)-9 or ICD-10 codes to define glaucomatous optic neuropathy, inconsistent and clear definitions of MetS, and analyses of data derived from participants at a single medical center. Therefore, further investigation is required to address these gaps and enhance understanding of the relationship between MetS and glaucoma.

The prevalence of glaucoma differs among various races and that of NTG is higher among Asians [19, 20]. Therefore, we aimed to determine whether MetS is associated with increased risk of glaucoma by analyzing data from a nationally representative survey in Korea.

## Methods

### Study population

In this retrospective, cross-section study, we analyzed all data from the 2019‒2021 Korean National Health and Nutrition Examination Survey (KNHANES). The KNHANES is a nationwide, representative, and population-based annual survey conducted by the Korea Disease Control and Prevention Agency (KDCA) that monitors the health and nutritional status of the Korean population [21]. The KNHANES adopts a three-stage sample design [22]. First, primary sample units (PSUs) are selected from a list of census blocks or resident registration addresses, with each PSU containing approximately 50–60 households. Second, 20 households are chosen through field surveys for household screening. Third, all members aged 1 year and above in the selected households participate in the survey. Each year, a total of approximately 10,000 individuals from 4800 households are sampled across all 192 PSUs. Over the 2019–2021 period, a total of 576 PSUs with 14,400 households are sampled for the KNHANES. The stratification variables are province, city/township/district, and housing type, while the implicit stratification variables were sex, age, residential area, and head of household's education level. Finally, sampling weights are assigned to each participant to generalize the units for representing the Korean population based on the estimated number of households and population for each year, so that the sum of household weights was equal to the total number of households in South Korea, and the sum of individual weights by survey category was equal to the population of the corresponding age group in South Korea [22]. Detailed information is available from the KNHANES website (https://knhanes.kdca.go.kr/knhanes/eng, accessed on May 8, 2023). 

**Fig. 1:**
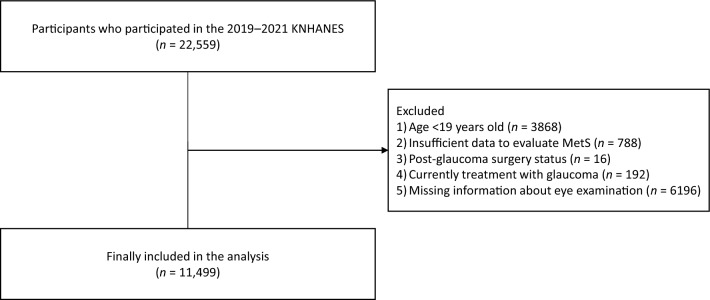
Flowchart of the study population selection. KNHANES: Korean National Health and Nutrition Examination Survey; MetS: metabolic syndrome

Figure 1 shows the flowchart of the study population selection. Among 22,559 patients listed in KNHANES 2019–2021, we excluded those aged < 19 y (*n* = 3868), insufficient data to evaluate MetS (*n* = 788), post-glaucoma surgery status (*n* = 16); currently under treatment for glaucoma (*n* = 192), and unknown glaucoma status (*n* = 6196). After excluding these, we analyzed data from 11,499 participants.

This study conformed to the ethical guidelines of the 1964 Declaration of Helsinki and its amendments. Written, informed consent was obtained from all eligible KNHANES participants. The Institutional Review Board (IRB) at Eulji University College of Medicine approved the study protocol (IRB number: 2022-07-022).

### Measurements

Height (cm) and weight (kg) were measured, then body mass index (BMI, kg/m^2^) was calculated. Waist circumference (cm) was measured at the midline between the lowest margin of the rib and highest margin of the iliac crest. Abdominal obesity was defined as waist circumference ≥ 90 (men) and ≥ 85 cm (women). Systolic (SBP) and diastolic (DBP) blood pressure (mmHg) was measured three times in seated participants at least 5 min after resting. The mean values of the last two measurements were defined as SBP and DBP. The mean blood pressure (MBP, mmHg) was calculated as DBP + 1/3 × (SBP − DBP). Cigarette use was classified as follows: nonsmoker (never smoked or had smoked < 100 cigarettes throughout life), ex-smoker (smoked ≥ 100 cigarettes throughout life but quit smoking by the time of the survey), or current smoker (smoked ≥ 100 cigarettes throughout life and had not quit by the time of the survey). Alcohol use was classified as currently, or not currently consumed. Physical activity was assessed using the Global Physical Activity Questionnaire [23], as the metabolic equivalent of task (MET) hours per day (MET-h/day). Participants were categorized into groups with low, moderate, or high physical activity (< 7.5, 7.5–30, and > 30 METs/h/day, respectively). Total energy intake was calculated using the 24-h recall method. Educational levels were categorized as elementary, middle, and high school, and college or university, and monthly household income was classified into quartiles. Fasting plasma glucose (FPG), serum total cholesterol, triglyceride, and high-density lipoprotein (HDL) cholesterol were measured in blood samples collected after fasting for at least 8 h.

To ensure quality control of ophthalmological exams and the survey, the KDCA and the Korean Ophthalmological Society (KOS) implemented team education and training programs twice a year. Four well-trained nurses were responsible for conducting visual field tests, IOP measurements, and optical coherence tomography (OCT) on the participants. All participants who aged 19 years or older underwent automated visual field examination employing frequency doubling technology (FDT), N30-5 screening, and Humphrey Matrix (Carl Zeiss Meditec) following standard protocols. Only reliable visual fields were included, characterized as having ≤ 1 fixation losses and false-positive responses. An abnormal visual field was denoted by the presence of at least one location exhibiting reduced sensitivity [24]. IOP was measured using the probe of an Icare PRO rebound tonometer (Icare Finland Oy, Helsinki, Finland) placed within 3–7 mm of the cornea, perpendicular to the central cornea in seated participants looking straight ahead. The IOP was measured by taking six consecutive measurements for each eye. The interpretation of the readings involved discarding the highest and lowest values among the six measurements and calculating the average of the remaining four readings [24]. To capture images of the fundus of the eye, non-mydriatic fundus photography (VISUCAM 224, Carl Zeiss Meditec) with a field angle of 45° was employed, which was reduced to 30° in cases of small pupils. Digital images were taken with physiological mydriasis in all participants. For each participant, one fundus image, covering both the macula and optic disc, was obtained for each eye. Additionally, red-free photography was acquired by digitally transforming the original color fundus photography [24]. Every participant in the study underwent a thorough examination of both the posterior and anterior segments using the Cirrus high-definition OCT (Cirrus HD-OCT 500, Carl Zeiss Meditec). Posterior segment imaging was obtained using the built-in program, which included the Macular Cube 512 × 128 images, two high-definition 5 Line Raster scans, and the Optic Disc Cube 200 × 200 protocol. Additionally, anterior segment imaging was conducted using the built-in 15.5 mm Wide Chamber View protocol [24].

### Definition of MetS

Using the diagnostic criteria for MetS based on the National Cholesterol Education Program Adult Treatment Panel III [25], we defined MetS when at least three of the following criteria were met: abdominal obesity, FPG ≥ 100 mg/dL or use of oral hypoglycemic agents or insulin therapy; serum triglycerides ≥ 150 mg/dL or under control with lipid-lowering agents; serum HDL cholesterol < 40 (men) and < 50 (women) mg/dL in women; SBP ≥ 130 mmHg, DBP ≥ 85 mmHg, or under control with anti-hypertensive agents. The participants were then assigned to groups with (*n* = 4384) and without (*n* = 7115) MetS.

### Definition of POAG and NTG

Using data from visual field test and OCT, POAG was defined according to the International Society for Geographic and Epidemiological Ophthalmology criteria [26]. A patient was diagnosed with POAG if they fell under Category I, II, or III.

Category I required both structural damage and corresponding visual field defects to be present together. The criteria included: (1) glaucomatous structural damage, such as thinning or notching of the optic disc, vertical cup-to-disc ratio of ≥ 0.7 (OCT), asymmetry of ≥ 0.2 between the two eyes, presence of retinal nerve fiber layer defect, or optic disc hemorrhage, with the location of the damage noted as superior, inferior, or none; and (2) glaucomatous visual field defects, defined as reduced sensitivity corresponding to retinal nerve fiber layer or optic disc abnormalities on the FDT perimetry test, with a fixation error and false positive rate ≤ 1, and the location of the defect noted as superior, inferior, or none.

Category II required clear structural damage without proven visual field defects, and the criteria included: (1) glaucomatous structural damage, such as thinning or notching of the optic disc with a vertical cup-to-disc ratio of ≥ 0.9, asymmetry of ≥ 0.3 between the two eyes, or presence of optic disc and corresponding retinal nerve fiber layer defects, with the location of the damage noted as superior, inferior, or none, and (2) absence of or fixation error and false positive rate ≥ 2 on FDT perimetry test.

Category III included patients who could not undergo visual field testing due to inability to observe the optic disc. Criteria included: (1) corrected visual acuity of less than 0.05 and IOP measured by rebound tonometry of ≥ 23 mmHg.

The KOS established a glaucoma reading committee, which consisted of 24 primary readers and 5 secondary readers in the first round of evaluations in 2019. In the second round, there were 21 primary readers and 6 secondary readers in 2020, and 36 primary readers and 6 secondary readers in the first round of evaluations in 2021. These readers reviewed the results of the visual field tests as well as OCT, and made diagnoses regarding the presence of glaucoma. Each patient's data was assessed by two primary readers, and they jointly made the initial assessment. The data was distributed among the readers based on regions, allowing them to review the relevant data for each specific week.

Finally, we defined ocular HTN as IOP > 21 mmHg and classified participants into POAG with ocular HTN group, and NTG (POAG without ocular HTN) group [27].

### Statistical analysis

Data are presented as means ± standard error (SE) for continuous variables and as ratios (% with SE) for categorical variables. Differences in continuous and categorical variables between groups with and without MetS were compared using weighted t-tests and chi-square tests, respectively.

We estimated ORs with 95% CI using univariate and multivariate weighted logistic regression analyses for POAG according to MetS status. Age, sex, and BMI were adjusted in Model 1. These adjusted variables plus socioeconomic factors and personal habits of total energy intake, smoking status, alcohol consumption, physical activity, education, and monthly household income were included in Model 2. The variables adjusted in model 2 plus metabolic factors, including MBP, FPG, and serum total cholesterol were included in Model 3. We further adjusted for IOP in Model 4 for sensitivity analysis.

We determined dose–response relationships between the number of MetS components and POAG. We created a cubic spline curve, then applied univariate and multivariate weighted logistic regression analyses for POAG per MetS incremental component.

All data were statistically analyzing using R (version 4.2.1; R Foundation for Statistical Computing, Vienna, Austria) and SAS statistical software (version 9.4; SAS Institute Inc., Cary, NC, USA). All tests were two-sided, and values with *p* < 0.05 were considered statistically significant.

## Results

### Clinical characteristics of the study population

Table 1 shows the clinical characteristics of the study population with and without MetS. The proportions of men, current smokers, and individuals with low levels of physical activity were significantly higher in the group with, than without MetS. They also had higher mean values for age, BMI, waist circumference, MBP, and a higher proportion of individuals in the lowest quartile of monthly household income and individuals with an elementary school education than those without MetS. Less participants consumed alcohol in the group with, than without MetS. Total energy intake between the groups did not significantly differ.

**Table 1:** Clinical characteristics of the study population with sampling weights stratified by metabolic syndrome status

Variables	Metabolic syndrome		p^*^
	No	Yes	
Unweighted number, n	7115	4384	
Male sex, % (SE)	44.9 (0.5)	57.1 (0.4)	< 0.001
Age, years	48.5 ± 0.5	57.3 ± 0.4	< 0.001
Body mass index, kg/m^2^	23.0 ± 0.0	26.5 ± 0.1	< 0.001
Waist circumference, cm	80.5 ± 0.1	92.3 ± 0.2	< 0.001
Mean blood pressure, mmHg	87.6 ± 0.2	95.6 ± 0.2	< 0.001
Smoking status, % (SE)			< 0.001
Non-smoker	16.9 (0.4)	22.3 (0.3)	
Ex-smoker	20.4 (0.4)	27.8 (0.3)	
Current smoker	62.7 (0.7)	49.9 (0.5)	
Currently drinking, % (SE)	51.3 (0.7)	45.5 (0.4)	< 0.001
Physical activity, % (SE)			< 0.001
Low (< 7.5 METs-hr/day)	48.6 (0.5)	58.2 (0.6)	
Moderate (7.5–30 METs-hr/day)	37.3 (0.6)	32.7 (0.4)	
High (> 30 METs-hr/day)	14.1 (0.4)	9.2 (0.2)	
Monthly household income, % (SE)			< 0.001
Lowest quartile	11.7 (0.4)	19.8 (0.4)	
Second quartile	21.6 (0.5)	25.3 (0.4)	
Third quartile	30.6 (0.6)	26.7 (0.4)	
Highest quartile	36.1 (1.0)	28.2 (0.4)	
Education level, % (SE)			< 0.001
Elementary school	7.8 (0.3)	20.8 (0.4)	
Middle school	6.8 (0.2)	11.8 (0.2)	
High school	38.3 (0.6)	35.4 (0.4)	
College/University	47.0 (0.9)	31.9 (0.4)	
Glucose, mg/dL	95.8 ± 0.2	115.6 ± 0.6	< 0.001
Total cholesterol, mg/dL	197.2 ± 0.6	189.4 ± 0.8	< 0.001
Total energy intake, kcal/day	1867.0 ± 14.1	1880.4 ± 19.4	0.556
Intraocular pressure, mmHg	15.3 ± 0.1	15.5 ± 0.1	< 0.001

*p-values were derived from a weighted t-test for continuous variables and a weighted chi-square test for categorical variables to compare the differences of each variable between groups

SE: standard error; METs: metabolic equivalents of task

**Fig. 2:**
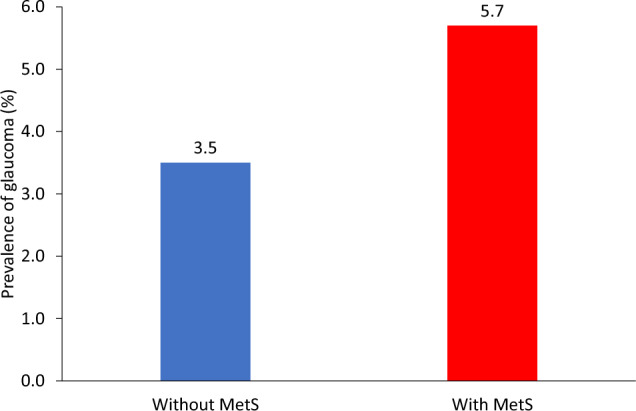
The prevalence of POAG according to the metabolic syndrome status. POAG: primary open-angle glaucoma

Figure 2 shows the prevalence of POAG according to MetS status. The prevalence of POAG was 5.7% (252 of 4384) and 3.5% (250 of 7115) patients with and without MetS, respectively.

### Association of MetS with risk of POAG

Table 2 shows the results of weighted logistic regression analysis of POAG risk according to MetS. The OR for POAG was higher in all models of the group with, than without MetS. The unadjusted OR was 1.85 (95% CI 1.52–2.25). The adjusted ORs (95% CI) for POAG in models 1, 2, 3 and 4 in the MetS group were 1.37 (1.04–1.80), 1.51 (1.10–2.08), 1.49 (1.04–2.13), and 1.47 (1.04–2.09), respectively. Risk for NTG was higher in patients with, than without MetS, whereas risk of POAG with ocular HTN did not significantly differ between the groups (Additional file 2: Table S1).

**Table 2:** Weighted logistic regression analysis for POAG according to the presence of metabolic syndrome

Risk of POAG	Metabolic syndrome		*p*
	No	Yes	
	OR	OR (95% CI)	
Unadjusted	1 (reference)	1.85 (1.52–2.25)	< 0.001
Model 1	1 (reference)	1.37 (1.04–1.80)	0.026
Model 2	1 (reference)	1.51 (1.10–2.08)	0.011
Model 3	1 (reference)	1.49 (1.04–2.13)	0.029
Model 4	1 (reference)	1.47 (1.04–2.09)	0.028

Model 1: adjusted for age, sex, and body mass index

Model 2: adjusted for variables used in Model 1 plus total energy intake, smoking status, drinking status, physical activity, education level, and monthly household income

Model 3: adjusted for variables used in Model 2 plus mean blood pressure, fasting plasma glucose level, and serum total cholesterol level

Model 4: adjusted for variables used in Model 3 plus intraocular pressure

POAG: primary open-angle glaucoma; OR: odds ratio; CI: confidence interval

### Dose–response relationships between MetS components and the risk of POAG

**Fig. 3:**
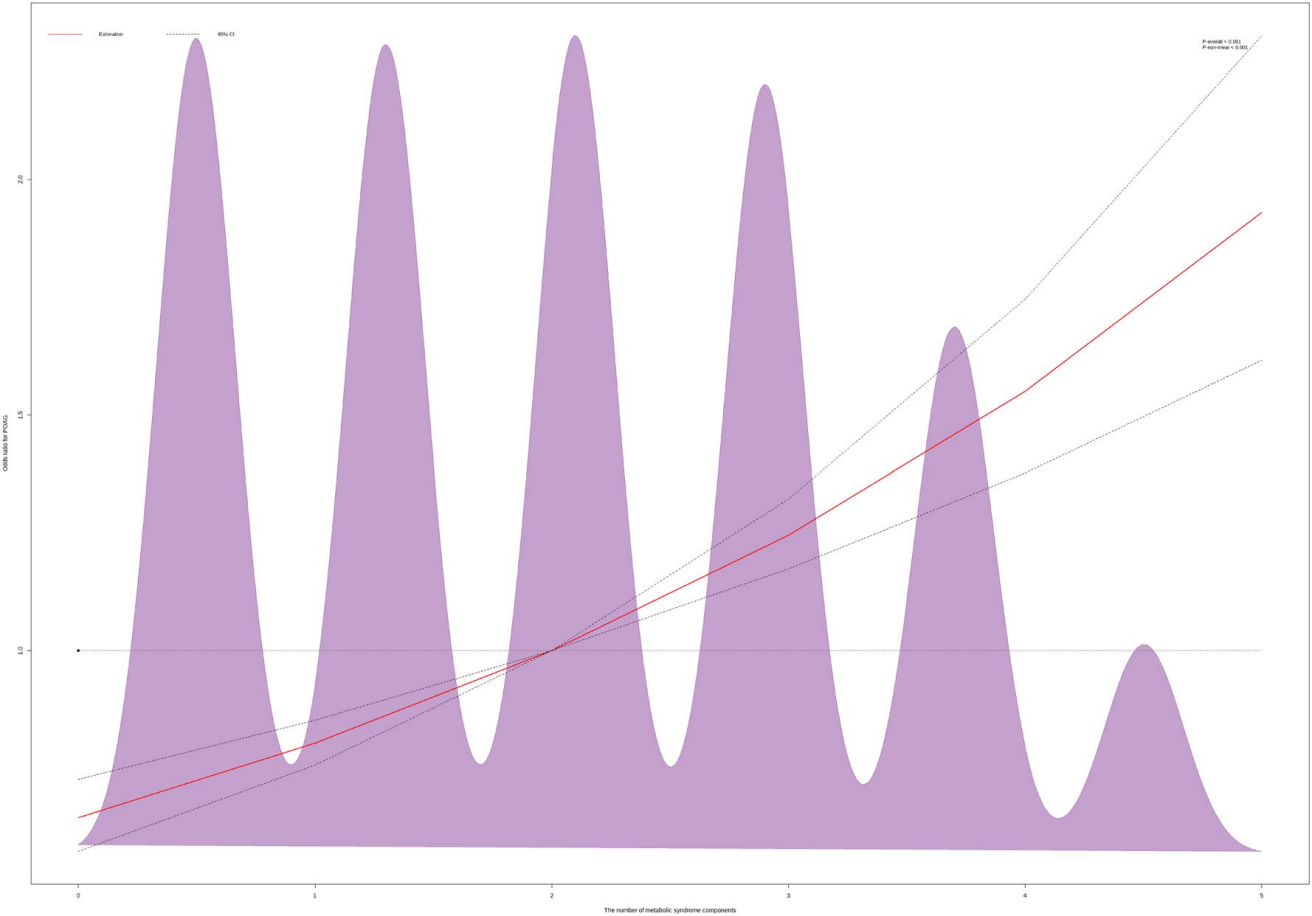
A cubic spline curve for hazard of POAG according to the number of metabolic syndrome components. POAG: primary open-angle glaucoma

The cubic spline curve in Fig. 3 shows that risk of POAG increased along with more MetS components. Table 3 shows the results of the weighted logistic regression analysis of POAG risk per increase in MetS components. The unadjusted OR was 1.28 (95% CI 1.19–1.38). The adjusted ORs (95% CI) for POAG per increment in the number of MetS components in models 1, 2, 3, and 4 were 1.17 (1.05–1.31), 1.21 (1.07–1.37), 1.19 (1.03–1.37), and 1.20 (1.04–1.39), respectively.

**Table 3:** Weighted logistic regression analysis for POAG risk by incremental number of metabolic syndrome components

Risk of POAG	Per increment in the number of metabolic syndrome components		
	OR	95% CI	*p*
Unadjusted	1.28	1.19–1.38	< 0.001
Model 1	1.17	1.05–1.31	0.005
Model 2	1.21	1.07–1.37	0.003
Model 3	1.19	1.03–1.37	0.020
Model 4	1.20	1.04–1.39	0.011

Model 1: adjusted for age, sex, and body mass index

Model 2: adjusted for variables used in Model 1 plus total energy intake, smoking status, drinking status, physical activity, education level, and monthly household income

Model 3: adjusted for variables used in Model 2 plus mean blood pressure, fasting plasma glucose level, and serum total cholesterol level

Model 4: adjusted for variables used in Model 3 plus intraocular pressure

POAG: primary open-angle glaucoma; OR: odds ratio; CI: confidence interval

### Association of MetS and its components with the risk of ocular HTN

Additional file 2: Table S2 shows the results of weighted logistic regression analysis of ocular HTN in patients with and without metabolic syndrome. The findings indicate that there is no significant relationship between MetS and ocular HTN. A significant dose–response relationship between the number of MetS components and risk of ocular HTN was not evident (Additional file 1: Fig. S1), however, the relationships were significant in univariable model (OR: 1.17; 95% CI 1.01–1.35) and multivariable models 1, 2, and 3, with adjusted ORs (95% CI) of 1.20 (1.00–1.44), 1.24 (1.03–1.51), and 1.21 (1.00–1.47), respectively for ocular HTN per increment in MetS components (Additional file 2: Table S3).

## Discussion

We found that MetS was significantly associated with an increased risk of POAG independently of IOP. Specifically, MetS and NTG were significantly associated, whereas MetS and POAG with ocular HTN were not. Notably, out of a total of 502 participants with POAG, only 19 (3.8%) participants had concurrent ocular HTN. This significant difference in the sizes between the NTG and POAG with ocular HTN groups might have contributed to disparate associations, even though sampling weights were applied to represent the general Korean population. Larger population-based studies are required to further understand this relationship.

The risk of POAG increased with more MetS components. A study of 18,240 adults aged ≥ 40 years at a single center positively associated the number of MetS components with risk for NTG (OR, 1.10), whereas and HTN and impaired glucose tolerance were significantly associated with NTG (OR, 1.53; 95% CI 1.20–1.94) and HTN (OR, 1.47; 95% CI 1.12–1.94 for impaired glucose tolerance) [28]. While one of the limitations in the previous study was the failure to identify a significant association between MetS and NTG stages, this study not only examined the relationship between MetS status and NTG but also explored its association with POAG with ocular HTN. Furthermore, the previous study's use of non-contact tonometry may have led to less precise IOP measurements, potentially resulting in an insignificant relationship between MetS and NTG. In contrast, our study utilized the rebound tonometer, which demonstrated higher correlation with the Goldmann Applanation tonometer, a gold standard method of IOP measurement, than the non-contact tonometer [29]. Lastly, our results represent the Korean population more accurately as we applied sampling weight to a Korean representative dataset. Consequently, we believe that we have overcome the limitations of the previous study.

While there was no significant association between ocular HTN and MetS, the risk of ocular HTN significantly increased with the incremental number of MetS components, even after adjusting for other confounding factors. Central corneal thickness plays a crucial role in determining IOP [30]. The risk of OHT is higher in people with, than without MetS, and this was attributed to a greater central corneal thickness in individuals with MetS [14, 15]. We could not include corneal thickness in analyses due to a lack of information. However, considering that Asians rank second to African Americans in terms of corneal thickness [31, 32], the effect of corneal thickness on ocular HTN in patients with MetS was attenuated in the present study.

Common features of MetS include insulin resistance, and oxidative and chronic low-grade inflammation [33], which can contribute to the development and progression of glaucoma. The death of RGCs, which are CNS neurons, is a crucial factor in the pathophysiology of all types of glaucoma [34]. Brain insulin resistance or central insulin signaling dysfunction is thought to contribute to transsynaptic neurodegeneration in glaucoma [35]. Central insulin resistance has been implicated in neurodegeneration due to its role in promoting neuroinflammation. The brain expresses abundant insulin and insulin-like growth factor-1 receptors, particularly in areas responsible for learning, memory, and neuroplasticity [36]. Insulin resistance disrupts the function of these receptors, resulting in impaired neuronal signaling and neuroinflammation [37]. These processes contribute to the death of RGCs and the development of glaucoma. Insulin resistance also negatively affects cerebral blood flow, induces vasculopathy, and worsens neurodegenerative processes [38]. Metabolic syndrome is characterized by peripheral insulin resistance, and it can also reflect central insulin resistance [39–41]. Toxic metabolites associated with MetS can activate various biochemical pathways, leading to oxidative stress and neuroinflammation, which in turn result in impaired insulin function in the brain (type 3 diabetes) [39]. Thioredoxin-interacting proteins are intracellular amplifiers of oxidative stress and inflammasome activation and could function in mediating central insulin resistance. Metabolic syndrome and insulin resistance have been linked to reduced cortical gray matter volume and thickness in a population-based investigation, but an independent effect of MetS on cortical gray matter was not detected beyond the impact of insulin resistance [40]. In this respect, the disrupted insulin signaling due to central insulin resistance in MetS might lead to neuroinflammation, vasculopathy, and ultimately the death of RGCs, thereby increasing the risk of glaucoma.

Oxidative stress and chronic inflammation resulting from imbalanced pro-oxidant and anti-oxidant processes, as well as activated inflammatory pathways are prevalent in individuals with MetS. These processes contribute to endothelial dysfunction and impaired ocular blood flow, both of which have been implicated in the pathogenesis of glaucoma. Moreover, oxidative stress and inflammation can directly damage ganglion cells and optic nerve fibers, further exacerbating glaucoma. Pro-inflammatory cytokines such as tumor necrosis factor (TNF)-α, interleukin-1 beta (IL-1β), and nitric oxide, secreted by activated glial cells, including microglia and astrocytes, can directly damage RGCs and optic nerve fibers. Tumor necrosis factor-alpha might directly act on RGCs to induce cell hyperexcitability by activating TNF receptor 1, which contributes to RGC apoptosis [42]. IL-1β can directly induce RGC death by promoting neuroinflammatory pathways, including the NLR family pyrin domain containing 3 inflammasome, mitogen-activated protein kinase, and nuclear factor kappa-light-chain-enhancer of activated B cells signaling [42, 43]. Nitric oxide can cause neurotoxicity by increasing oxidative stress and inducing mitochondrial dysfunction in RGCs [44].

This study has several limitations. First, information regarding corneal thickness and anterior chamber angles was insufficient. In addition, it should be noted that we were unable to evaluate the information related to intra- and inter-rater reliability, despite the affirmation by the KOS epidemiologic survey committee that they had verified the quality of the ophthalmic survey [24]. Second, the severity of POAG was not considered. Furthermore, we were unable to determine the exact number of individuals excluded due to unreliable visual field tests. Third, a causal relationship between MetS and POAG could not be verified due to the cross-sectional design of the study. Finally, our results cannot be applied to other ethnic groups because we only analyzed representative data from one nation. Nevertheless, we applied sampling weights to representative Korean data and confirmed that the association between MetS and POAG was particularly significant in NTG. We also identified a dose–response relationship between risk of POAG and the number of MetS components.

## Conclusions

Persons with MetS in the general Korean population are at increased risk of developing POAG, particularly NTG. A significant relationship was maintained regardless of IOP. Our findings indicated the importance of considering POAG as an ocular comorbidity in patients with MetS. Further investigation is required to determine whether changes in MetS status affect the development and progression of POAG.

### Supplementary Information

**Additional file 1:** Dose-response relationship between the number of MetS components and risk of ocular HTN.

**Additional file 2:** **Table S1. **Weighted logistic regression analysis for the risk of normal tension glaucoma and POAG with ocular hypertension according to the presence of metabolic syndrome. **Table S2.** Weighted logistic regression analysis for ocular hypertension according to the presence of metabolic syndrome. **Table S3.** Weighted logistic regression analysis for ocular hypertension risk by incremental number of metabolic syndrome components.

## Data Availability

The KNHANES data are available by registering on the official KNHANES website (https://knhanes.kdca.go.kr/knhanes/eng, accessed on May 8, 2023).

## Abbreviations

BMI: Body mass index
CI: Confidence interval
CNS: Central nervous system
DBP: Diastolic blood pressure
FPG: Fasting plasma glucose
HDL: High-density lipoprotein
HTN: Hypertension
ICD: International classification of diseases
IL-1β: Interleukin-1 beta
IOP: Intraocular pressure
IRB: Institutional review board
KNHANES: Korean National Health and Nutrition Examination Survey
MBP: Mean blood pressure
MET: Metabolic equivalent of task
MetS: Metabolic syndrome
NTG: Normal tension glaucoma
OR: Odds ratio
POAG: Primary open-angle glaucoma
RGCs: Retinal ganglion cells
SBP: Systolic blood pressure
SE: Standard error
TNF-α: Tumor necrosis factor-alpha
