# Supplementary figures and images for: Metabolic syndrome as an independent risk factor for glaucoma: a nationally representative study
Source: Diabetol Metab Syndr. 2023 Aug 24;15:177. doi: 10.1186/s13098-023-01151-5 (PMC10464157; doi:10.1186/s13098-023-01151-5)

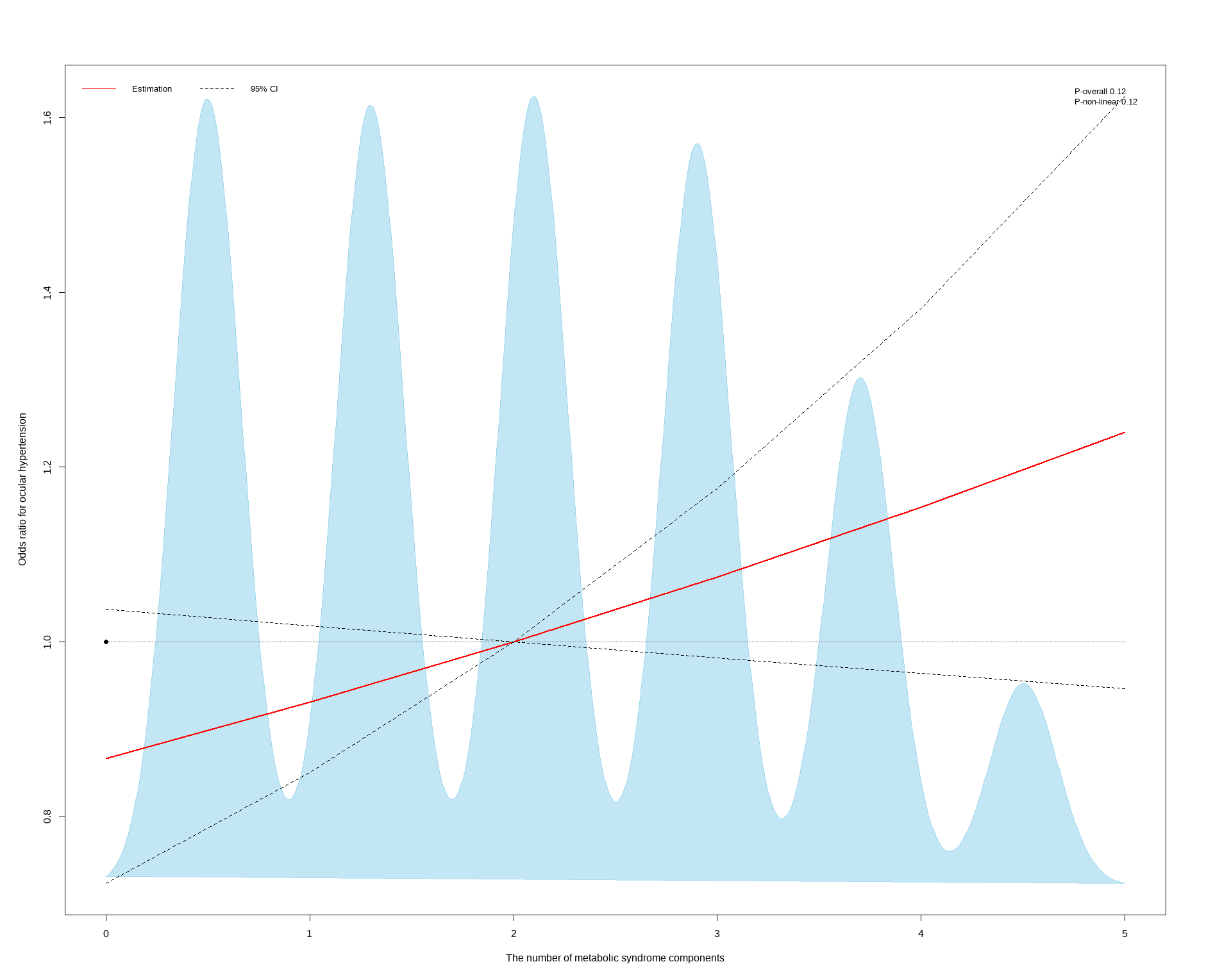

Supplement: Supplementary file 1 — Additional file 1: Dose-response relationship between the number of MetS components and risk of ocular HTN. [file 13098_2023_1151_MOESM1_ESM.tiff]
