# Supplementary material for: Metabolic syndrome as an independent risk factor for glaucoma: a nationally representative study
Source: Diabetol Metab Syndr. 2023 Aug 24;15:177. doi: 10.1186/s13098-023-01151-5 (PMC10464157; doi:10.1186/s13098-023-01151-5)
Supplement: Supplementary file 2 — Additional file 2: Table S1. Weighted logistic regression analysis for the risk of normal tension glaucoma and POAG with ocular hypertension according to the presence of metabolic syndrome. Table S2. Weighted logistic regression analysis for ocular hypertension according to the presence of metabolic syndrome. Table S3. Weighted logistic regression analysis for ocular hypertension risk by incremental number of metabolic syndrome components. [file 13098_2023_1151_MOESM2_ESM.docx]

**Table S1.** Weighted logistic regression analysis for the risk of normal tension glaucoma and POAG with ocular hypertension according to the presence of metabolic syndrome.

|  | Metabolic syndrome |  |  |
| --- | --- | --- | --- |
|  | No | Yes |  |
|  | OR | OR (95% CI) | *p* |
| NTG vs. normal |  |  |  |
| Unadjusted | 1 (reference) | 1.84 (1.51–2.25) | <0.001 |
| Model 1 | 1 (reference) | 1.41 (1.07–1.86) | 0.014 |
| Model 2 | 1 (reference) | 1.53 (1.11–2.11) | 0.010 |
| Model 3 | 1 (reference) | 1.50 (1.05–2.15) | 0.027 |
| POAG with ocular hypertension^*^ vs. normal |  |  |  |
| Unadjusted | 1 (reference) | 2.63 (0.94–7.38) | 0.066 |
| Model 1 | 1 (reference) | 0.91 (0.23–3.64) | 0.890 |
| Model 2 | 1 (reference) | 0.72 (0.12–4.49) | 0.724 |
| Model 3 | 1 (reference) | 0.84 (0.12–5.68) | 0.854 |

^*^Ocular hypertension was defined as intraocular pressure >21 mmHg.

Model 1: adjusted for age, sex, and body mass index.

Model 2: adjusted for variables used in Model 1 plus total energy intake, smoking status, drinking status, physical activity, education level, and monthly household income.

Model 3: adjusted for variables used in Model 2 plus mean blood pressure, fasting plasma glucose level, and serum total cholesterol level.

Abbreviations: NTG, normal tension glaucoma; POAG, primary open-angle glaucoma; OR, odds ratio; CI, confidence interval.

**Table S2.** Weighted logistic regression analysis for ocular hypertension according to the presence of metabolic syndrome.

|  | Metabolic syndrome |  |  |
| --- | --- | --- | --- |
|  | No | Yes |  |
| Risk of ocular hypertension^*^ | OR | OR (95% CI) | *p* |
| Unadjusted | 1 (reference) | 1.45 (0.96–2.18) | 0.077 |
| Model 1 | 1 (reference) | 1.43 (0.83–2.44) | 0.195 |
| Model 2 | 1 (reference) | 1.69 (0.96–3.00) | 0.072 |
| Model 3 | 1 (reference) | 1.52 (0.86–2.70) | 0.151 |

^*^Ocular hypertension was defined as intraocular pressure >21 mmHg.

Model 1: adjusted for age, sex, and body mass index.

Model 2: adjusted for variables used in Model 1 plus total energy intake, smoking status, drinking status, physical activity, education level, and monthly household income.

Model 3: adjusted for variables used in Model 2 plus mean blood pressure, fasting plasma glucose level, and serum total cholesterol level.

Abbreviations: OR, odds ratio; CI, confidence interval.

**Table S3.** Weighted logistic regression analysis for ocular hypertension risk by incremental number of metabolic syndrome components

|  | Per increment in the number of metabolic syndrome components | | |
| --- | --- | --- | --- |
| Risk of ocular hypertension^*^ | OR | 95% CI | *p* |
| Unadjusted | 1.17 | 1.01–1.35 | 0.036 |
| Model 1 | 1.20 | 1.00–1.44 | 0.046 |
| Model 2 | 1.24 | 1.03–1.51 | 0.027 |
| Model 3 | 1.21 | 1.00–1.47 | 0.049 |

^*^Ocular hypertension was defined as intraocular pressure >21 mmHg.

Model 1: adjusted for age, sex, and body mass index.

Model 2: adjusted for variables used in Model 1 plus total energy intake, smoking status, drinking status, physical activity, education level, and monthly household income.

Model 3: adjusted for variables used in Model 2 plus mean blood pressure, fasting plasma glucose level, and serum total cholesterol level.

Abbreviations: OR, odds ratio; CI, confidence interval.
